# A Thursday Afternoon Clinique at the London Polyclinic

**Published:** 1906-11-10

**Authors:** Jonathan Hutchinson


### Hospital Clinics.

### A THURSDAY AFTERNOON CLINIQUE AT THE LONDON POLYCLINIC.

By Mr. JONATHAN HUTCHINSON, F.R.C.S.Eng., LL.D., F.R.S., etc.

The afternoon of Thursday attracts to this College a more than ordinary crowd of practitioners from far and near to witness the methods of and to hear at his work one of the most distinguished ornaments of British medical science of the day. On Thursday last Mr. Hutchinson, fresh from the honour of having received the Moxon Medal, was happy in consultation with fellow-practitioners over perplexing cases brought forward by them for his diagnosis and clinical opinion. His clinique is always invested with the charm of variety and interest, full of suggestion and mental stimulus.

### A Doubtful Abdominal Tumour.

The first case presented a swelling over the region of the gall-bladder. The mother of the patient had recently died of malignant growth, but the patient showed no symptoms of malignancy. The swelling had not increased in growth for eleven months, it moved quite easily, and was situated in the region of the gall-bladder. There was no evidence of enlargement of the liver or of other liver disease. Against the supposition that the swelling was a fibroid growth in the omentum it was not multiple, and the patient complained of pain. There were no symptoms of gall-stone. The swelling was close to the abdominal wall, and on pressure it insisted upon coming forwards. It was not attached to the abdominal wall, but moved as if it were a pendulous attachment of the liver. The tumour was within the abdomen. Hydatid cyst was considered, but no fluctuation could be elicited, and the small size of the swelling precluded that. Captain Pinch made the ingenious suggestion that it might be a diverticulum of the gall-bladder. Mr. Hutchinson thought the balance of evidence indicated the gall-bladder, but observed that he should not like to give a positive opinion, as he had never found a gall-bladder coming so easily forward. On the whole, however, his opinion gravitated towards the gall-bladder.

### Infantile Paralysis.

A child was shown with a contraction of the left foot probably due to infantile paralysis. There was wasting of the limb and contraction of the sole of the foot. Mr. Hutchinson discussed the kind of boot that should be worn, and indicated the employment of electricity and friction. In such cases, he observed, the plantar facia was contracted and might be cut, but the tendons should be avoided. Cutting tendons in infantile paralysis frequently left much weakening. He would not be in any hurry about operating in connection with this case. The boot and the use of electricity and friction would probably improve the condition somewhat. There was no history of other muscles having been affected.

### Ubi Irritatio ibi Fluxus.

Mr. Hutchinson showed specimens of dead beech leaves with patches of localised greening still remaining. At this time of the year leaves have run through their summer functions and are becoming senile, beginning to have feelings of old age. They become brown, the leaf gets detached, and falls. Under a beech tree one finds about one in every five hundred of these dead brown leaves streaked with broad patches of green. The question is, What keeps the patch green? The condition illustrates a well-known law, both in animal and vegetable physiology. The explanation is that upon the leaf a number of galls have grown, looking like warts, probably due to deposition of the egg of a living insect. The galls draw their nourishment from the leaf sap and are not subject to the necessity of senile death during the autumn as the leaf is. This fact suffices to attract the sap to the part, and it becomes diffused over the whole leaf or over a large part of it, so that the leaf gains from having in a friendly manner harboured this parasite. This leaf affords a very interesting demonstration that "where there is irritation there is a flow," *ubi irritatio ibi fluxus*. The irritation of the insect suffices to protect the vitality of the leaf, or of parts of the leaf, for some weeks or even months. The greenness is not only adjacent to the gall, but extends over a large portion, and sometimes is distally situated as regards the gall, affording a demonstration of a *vis a fronte* action being concerned in the circulation in vegetables, and therefore in animals. Numerous examples of this are found in the human body, for example, the irritation of cancer, attracting blood to the affected and neighbouring parts, causing local irritation and a certain amount of hypertrophy and overgrowth. Every leaf that keeps green beyond its time has some disease in it.

### Malignant Disease of the Larynx.

The next patient had congenital cleft palate, had lost his voice since last Easter, the glands of his neck were considerably swollen, and he suffered with cough. The large glandular swelling behind the ear involved the sterno-cleido-mastoid, and Mr. Hutchinson alleged that it might be secondary to some epitheliomatous disease of the larynx. This view obtained support from induration of the glands situated lower down the neck. The glands in this case had taken on a character of extreme induration, a number of them hanging together and leaving the impression of being due to some primary disease in the larynx. Mr. Hutchinson's view was that it was a case of malignant disease of the larynx beginning at the base of the epiglottis. No pain was present either in the throat or in the ear. The case was referred to Dr. Tilley for laryngeal examination.

Leucoderma.

Mr. Hutchinson showed the portrait of a case of leucoderma—definite circular white patches on the body, sometimes on the scalp, not involving any condition of disturbance of the general health, and occurring in youngish people.

It resembles in some respects alopecia areata, a disease in which the loss of hair on the scalp occurs in patches, but the causation of which is surrounded with great obscurity. Mr. Hutchinson suggested the theory, that these conditions were sequelæ of ringworm. Many cases have been observed by him in which evidence of a previous attack of ringworm had been present years before the alopecic condition occurred. In alopecia areata the hair will grow again, on the application of chrysophanic ointment, but very often it grows white, even blanched over the whole of the part which was affected, and sometimes the condition tends to spread. It is very rarely cured as regards the skin reproducing its pigment. Mr. Hutchinson mentioned the case of two brothers, young men, one with large patches of almost snowy white hair on his scalp, showing strongly amongst his black hair and looking as if the hair would ultimately become blanched. On various parts of his body white patches were also found. The other brother had alopecia areata, a destruction of the hair, and not merely a destruction of the pigment. The two had a certain amount of similarity. Both had had ringworm, and Mr. Hutchinson thought that the conditions might be due to alteration of the character of the ringworm disease. If a parasite could be found, the whole thing would be clear. Many persons have searched diligently for a parasitic explanation, but it has never been found. Mr. Hutchinson suggests that the association between ringworm and alopecia areata may be due to a modification of the fungus which separately produced each disease. Science cannot disprove the suggestion that the bacillus of tuberculosis may be changed by special feeding into the bacillus of leprosy. Many forms of fungal life are changed by their food and environment. So true are fungi to their food supply that the name of the animal may be predicated from the growth of certain fungi on its dung. We have not got to the limit of knowledge as to the lives of these fungal forms. He suggested that the tubercle bacillus can change into the bacillus of leprosy by altering its feeding and its environment. The two are exceedingly difficult to distinguish and are very closely allied. It is probable that the bacillus of leprosy is not present in fish, but some stimulating element which is capable of differentiating them may be present. Minute differences between the two must be admitted, but in some cases it is most difficult to determine which is which, and the difference may be due to a different kind of food. One is obliged to speculate about things one does not understand, and it might be possible that these parasites usually associate themselves with one or other tissue, and that they then pursue a life of partnership, as in the case of the lichen, which is produced by an alga and a fungus—the one a true fungus, the other a true seaweed, growing together in partnership and producing what is termed a lichen. A similar partnership may exist between the tubercle bacillus and the bacillus of leprosy, and Mr. Hutchinson believed that the different forms of lupus could best be explained by considering that a kind of partnership is capable of being formed between the parasite and the cell-element in which it lives.

